# Takotsubo Syndrome Associated with ST Elevation Myocardial Infarction

**DOI:** 10.1155/2019/1010243

**Published:** 2019-05-16

**Authors:** Saad Ezad, Michael McGee, Andrew J. Boyle

**Affiliations:** ^1^John Hunter Hospital, Hunter New England Health, Newcastle, NSW, Australia; ^2^The University of Newcastle, Newcastle, NSW, Australia; ^3^Hunter Medical Research Institute, Newcastle, NSW, Australia

## Abstract

**Background:**

Takotsubo syndrome is a reversible heart failure syndrome which often presents with symptoms and ECG changes that mimic an acute myocardial infarction. Obstructive coronary artery disease has traditionally been seen as exclusion criteria for the diagnosis of takotsubo; however, recent reports have called this into question and suggest that the two conditions may coexist.

**Case Summary:**

We describe a case of an 83-year-old male presenting with chest pain consistent with acute myocardial infarction. The ECG demonstrated anterior ST elevation with bedside echocardiography showing apical wall motion abnormalities. Cardiac catheterisation found an occluded OM2 branch of the left circumflex artery with ventriculography confirming apical ballooning consistent with takotsubo and not in the vascular territory supplied by the occluded epicardial vessel. Repeat echocardiogram 6 weeks later confirmed resolution of the apical wall motion abnormalities consistent with a diagnosis of takotsubo.

**Discussion:**

This case demonstrates the finding of takotsubo syndrome in a male patient with acute myocardial infarction. Traditionally, this would preclude a diagnosis of takotsubo; however, following previous reports of takotsubo in association with coronary artery dissection and acute myocardial infarction in female patients, new diagnostic criteria have been proposed which allow the diagnosis of takotsubo in the presence of obstructive coronary artery disease. This case adds to the growing body of literature that suggests takotsubo can coexist with acute myocardial infarction; however, it remains to be elucidated if it is a consequence or cause of myocardial infarction.

## 1. Introduction

First described in a case series of Japanese patients in 1991 [1], takotsubo syndrome (TTS) is a rapidly reversible heart failure syndrome most commonly seen in postmenopausal women following emotional or physical stress [2]. Various terms have been used to describe this condition including broken heart syndrome [3], takotsubo cardiomyopathy [4], and stress-induced cardiomyopathy [5]. TTS is now the preferred nomenclature as patients with takotsubo do not appear to have primary muscle pathology [6]. Presentation mimics acute myocardial infarction (AMI) with chest pain and dyspnoea often associated with ST segment elevation or T wave inversion. In a contemporary western population, an estimated 0.9% of patients admitted for primary PCI were diagnosed with TTS [7]. However, data from the International Takotsubo Registry has shown coronary artery disease coexists in 15.3% of patients with a diagnosis of TTS [8]. The underlying pathophysiology has yet to be fully understood, although a rapid elevation in circulating catecholamine levels in response to stress has traditionally been believed to be a central feature [9]. More recently, however, it has been demonstrated that plasma catecholamine levels are normal or only mild-moderately elevated in patients with TTS [10] and that local cardiac sympathetic hyperactivation results in myocardial stunning [11].

## 2. Case Presentation

**Figure 1:**
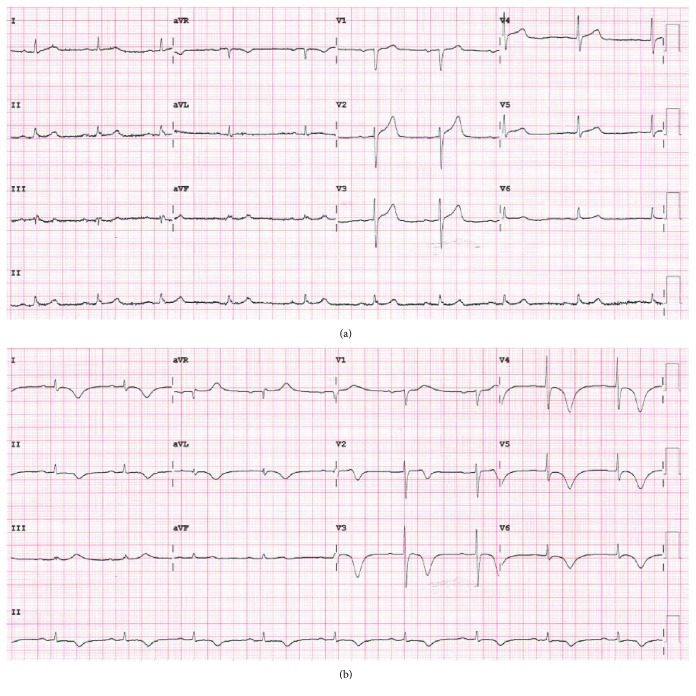
(a) Admission ECG showing minimal anterior ST elevation and concave ST segments in leads II, III, and aVF. (b) ECG 48 hours after presentation demonstrating deep T wave inversion across precordial and limb leads with a prolonged QTc.

An 83-year-old gentleman with a past medical history of diet-controlled diabetes mellitus type 2, gout, and hypertension presented to our institution with a 4-hour history of upper abdominal pain and lower chest tightness associated with dyspnoea, which was partially relieved by intravenous morphine and sublingual glyceryl trinitrate administered by ambulance paramedics. On arrival in the emergency department, a 12-lead ECG showed minimal anterior ST elevation (Figure 1(a)); therefore, a bedside echocardiogram was performed. This demonstrated hypokinesis of the apical third of the anterior, inferior, and lateral walls. Given the borderline ECG changes and regional wall motion abnormalities on echo, the patient was taken for emergency cardiac catheterisation.

**Figure 2:**
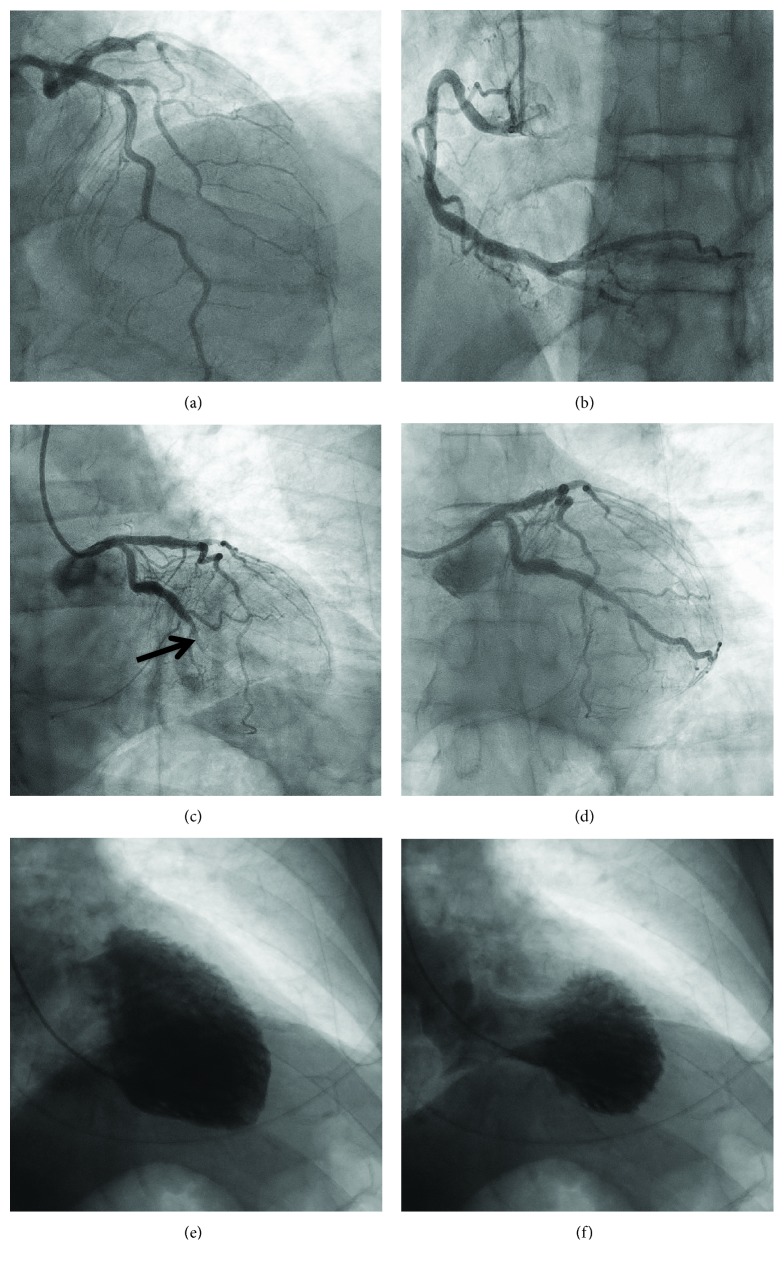
Coronary angiography on admission. (a) Minor disease in the left anterior descending artery. (b) Mild-moderate disease in the right coronary artery. (c) Occluded OM2 branch (arrow) of the left circumflex artery. (d) TIMI III flow post percutaneous coronary intervention (PCI) with drug-eluting stent (DES). Left ventriculogram in the right anterior oblique (RAO) projection during diastole (e) and systole (f) showing mid apical pattern of takotsubo syndrome with sparing of the apical tip.

Angiography revealed an occluded obtuse marginal 2 (OM2) branch of the circumflex artery (Figure 2(c)) with minor disease in the other major epicardial arteries (Figures 2(a) and 2(b)). Flow was restored following passage of the guidewire, and thrombus was clearly identifiable in the vessel. The lesion was treated with one 2.5 mm × 15 mm drug-eluting stent resulting in TIMI III flow (Figure 2(d)).

Ventriculogram done in the RAO projection revealed mid and apical hypokinesis and ballooning with preserved basal function (Figures 2(e) and 2(f)). Ventriculogram from the LAO projection showed posterior wall hypokinesis more in keeping with the ischaemic territory affected by acute plaque rupture.

A venous blood gas revealed haemoglobin of 145 g/L (ref 120-170 g/L), normal electrolytes, and blood glucose of 8.7 mmol/L (ref 3.5-7.7 mmol/L). The patient's initial troponin I was 365 ng/L (ref <26 ng/L) and peaked at 17,180 ng/L the following day. His ECG evolved to show deep symmetrical T wave inversion across the anterolateral and limb leads, clearly more extensive than the distribution of the infarct artery (Figure 1(b)) associated with the prolongation of the QT interval. Formal echocardiogram performed 6 hours following percutaneous coronary intervention (PCI) showed severe apical ballooning and hypokinesis extending to mid cavity with preservation of basal function, consistent with TTS. The posterolateral wall was also noted to be akinetic in keeping with a region of infarction. There was mild LV systolic dysfunction (EF 45%).

**Figure 3:**
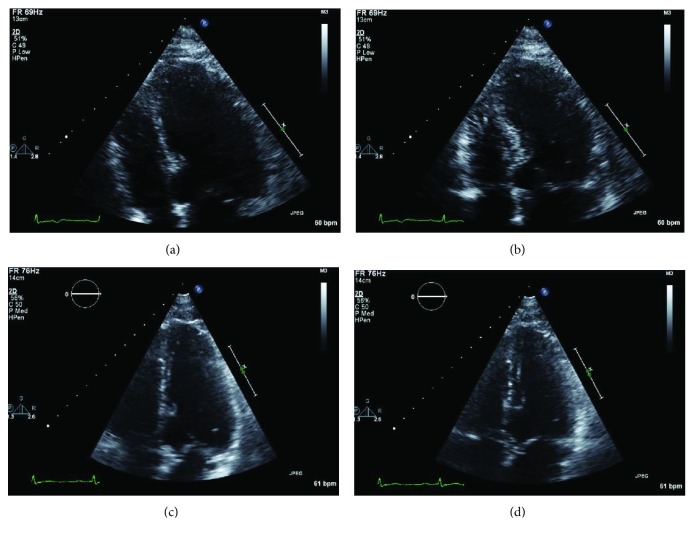
Transthoracic echocardiogram on admission and 6-week follow-up. Apical 4 chamber window at end-diastole (a) and at end-systole (b) showing apical dilatation during acute presentation. (c) Apical 4 chamber window at end-diastole at 6-week follow-up. (d) Apical 4 chamber window at end-systole at 6-week follow-up revealing resolution of apical ballooning.

The patient was commenced on perindopril and atorvastatin in addition to dual antiplatelet therapy with aspirin and clopidogrel. On further questioning, no acute emotional triggers in the patient's life could be identified. On day 3 of the patient's admission, troponin was down trending at 8907 ng/L. He was discharged 4 days after presentation, following an uncomplicated inpatient stay. Follow-up echocardiography performed 6 weeks after discharge demonstrated restoration of normal LV systolic function and resolution of the previously seen regional wall motion abnormalities (Figure 3).

## 3. Discussion

The current Mayo diagnostic criteria [12] require the presence of a transient regional wall motional abnormality, which extends beyond a single epicardial vascular distribution, and the absence of obstructive coronary artery disease or evidence of acute plaque rupture. However, several recent reports of TTS in association with coronary artery dissection [13] and acute myocardial infarction [14, 15] have led to newly proposed diagnostic criteria which state that TTS may exist as a comorbidity with a variety of illnesses including acute coronary syndromes [16].

To our knowledge, this is a unique case describing the association of acute myocardial infarction (AMI) caused by acute plaque rupture and TTS in a male patient. The lack of an emotional trigger in this case suggests a possible causal relationship between the two conditions. The stress associated with AMI could conceivably have resulted in sympathetic activation causing TTS in a distribution not perfused by the occluded OM2 artery. Alternatively, postischaemic myocardial stunning has been proposed as a possible trigger factor [17]. Conversely, AMI may be triggered by TTS [18]. A recent report described a case of thromboembolism from a left ventricular thrombus as a result of TTS causing AMI [19]. Alternatively, pain has been described as a trigger for TTS [20], and the chest pain from AMI could potentially have triggered sympathetic activation.

Frangieh et al. found that T wave inversion on presentation was twice as common in TTS (45%) compared with AMI (22%) [21]. The lack of T wave inversion on presentation in this case followed by the development of deep T wave inversion across the precordial leads could suggest that AMI was the initial pathology followed by TTS. Furthermore, the QTc interval increased from 390 msec on the presentation to 527 msec when the T wave inversion evolved. A prolonged QT interval has also been associated with TTS rather than AMI [21, 22]. A shorter time to peak troponin (<6 hours) and smaller troponin rise have been found to be predictive of TTS [22], in contrast to our case where a large troponin rise was seen which peaked 24 hours after admission.

This case adds to the growing body of literature suggesting TTS and coronary artery disease may not be mutually exclusive as once thought; however, more work is required to identify the nature of the link and whether TTS is the cause or consequence of AMI in those patients in whom it coexists.

## References

[1] Dote K., Sato H., Tateishi H., Uchida T., Ishihara M. (1991). Myocardial stunning due to simultaneous multivessel coronary spasms: a review of 5 cases. *Journal of Cardiology*.

[2] Bossone E., Savarese G., Ferrara F. (2013). Takotsubo cardiomyopathy: overview. *Heart Failure Clinics*.

[3] Mukherjee A., Sunkel-Laing B., Dewhurst N. (2013). ‘Broken heart’ syndrome in Scotland: a case of Takotsubo cardiomyopathy in a recently widowed lady. *Scottish Medical Journal*.

[4] Sharkey S. W., Lesser J. R., Maron M. S., Maron B. J. (2011). Why not just call it tako-tsubo cardiomyopathy: a discussion of nomenclature. *Journal of the American College of Cardiology*.

[5] Maron B. J., Towbin J. A., Thiene G. (2006). Contemporary definitions and classification of the cardiomyopathies: an American Heart Association Scientific Statement from the Council on Clinical Cardiology, Heart Failure and Transplantation Committee; Quality of Care and Outcomes Research and Functional Genomics and Translational Biology Interdisciplinary Working Groups; and Council on Epidemiology and Prevention. *Circulation*.

[6] Lyon A. R., Bossone E., Schneider B. (2016). Current state of knowledge on takotsubo syndrome: a position statement from the taskforce on takotsubo syndrome of the Heart Failure Association of the European Society of Cardiology. *European Journal of Heart Failure*.

[7] Showkathali R., Patel H., Ramoutar A. (2014). Typical takotsubo cardiomyopathy in suspected ST elevation myocardial infarction patients admitted for primary percutaneous coronary intervention. *European Journal of Internal Medicine*.

[8] Templin C., Ghadri J. R., Diekmann J. (2015). Clinical features and outcomes of takotsubo (stress) cardiomyopathy. *The New England Journal of Medicine*.

[9] Wittstein I. S., Thiemann D. R., Lima J. A. C. (2005). Neurohumoral features of myocardial stunning due to sudden emotional stress. *The New England Journal of Medicine*.

[10] Y-Hassan S. (2018). Plasma epinephrine levels and its causal link to takotsubo syndrome revisited: critical review with a diverse conclusion. *Cardiovascular Revascularization Medicine*.

[11] Y-Hassan S., De Palma R. (2017). Contemporary review on the pathogenesis of takotsubo syndrome: the heart shedding tears. Norepinephrine churn and foam at the cardiac sympathetic nerve terminals. *International Journal of Cardiology*.

[12] Prasad A., Lerman A., Rihal C. S. (2008). Apical ballooning syndrome (Tako-Tsubo or stress cardiomyopathy): a mimic of acute myocardial infarction. *American Heart Journal*.

[13] Y-Hassan S., Henareh L. (2013). Spontaneous coronary artery dissection triggered post-ischemic myocardial stunning and takotsubo syndrome: two different names for the same condition. *Cardiovascular Revascularization Medicine*.

[14] Hurtado Rendón I. S., Alcivar D., Rodriguez-Escudero J. P., Silver K. (2018). Acute myocardial infarction and stress cardiomyopathy are not mutually exclusive. *The American Journal of Medicine*.

[15] Abreu G., Rocha S., Bettencourt N. (2016). An unusual trigger causing takotsubo syndrome. *International Journal of Cardiology*.

[16] Madias J. E. (2014). Why the current diagnostic criteria of takotsubo syndrome are outmoded: a proposal for new criteria. *International Journal of Cardiology*.

[17] Y-Hassan S., Themudo R., Maret E. (2017). Spontaneous coronary artery dissection and takotsubo syndrome: the chicken or the egg causality dilemma. *Catheterization and Cardiovascular Interventions*.

[18] Madias J. E. (2015). On a plausible association of spontaneous coronary artery dissection and takotsubo syndrome. *Canadian Journal of Cardiology*.

[19] Y-Hassan S., Holmin S., Abdula G., Böhm F. (2019). Thrombo-embolic complications in takotsubo syndrome: review and demonstration of an illustrative case. *Clinical Cardiology*.

[20] Daly M. J., Dixon L. J. (2010). Takotsubo cardiomyopathy in two preoperative patients with pain. *Anesthesia & Analgesia*.

[21] Frangieh A. H., Obeid S., Ghadri J. R. (2016). ECG criteria to differentiate between takotsubo (stress) cardiomyopathy and myocardial infarction. *Journal of the American Heart Association*.

[22] Vaidya G., Jaiswal A. J., Madhira B. (2016). ‘GET QT’: clinical criteria to differentiate takotsubo cardiomyopathy from STEMI. *International Cardiovascular Forum Journal*.

